# COVID-19 outbreak response, a dataset to assess mobility changes in Italy following national lockdown

**DOI:** 10.1038/s41597-020-00575-2

**Published:** 2020-07-08

**Authors:** Emanuele Pepe, Paolo Bajardi, Laetitia Gauvin, Filippo Privitera, Brennan Lake, Ciro Cattuto, Michele Tizzoni

**Affiliations:** 1 ISI Foundation, via Chisola 5, Turin, 10126 Italy; 2 Cuebiq Inc., New York, NY USA; 3 University of Turin, Turin, Italy

**Keywords:** Epidemiology, Viral infection, Lifestyle modification

## Abstract

Italy has been severely affected by the COVID-19 pandemic, reporting the highest death toll in Europe as of April 2020. Following the identification of the first infections, on February 21, 2020, national authorities have put in place an increasing number of restrictions aimed at containing the outbreak and delaying the epidemic peak. On March 12, the government imposed a national lockdown. To aid the evaluation of the impact of interventions, we present daily time-series of three different aggregated mobility metrics: the origin-destination movements between Italian provinces, the radius of gyration, and the average degree of a spatial proximity network. All metrics were computed by processing a large-scale dataset of anonymously shared positions of about 170,000 de-identified smartphone users before and during the outbreak, at the sub-national scale. This dataset can help to monitor the impact of the lockdown on the epidemic trajectory and inform future public health decision making.

## Abstract (summary)

**Table:** 

Measurement(s)	mobility
Technology Type(s)	GPS navigation system
Factor Type(s)	temporal interval
Sample Characteristic - Organism	Homo sapiens
Sample Characteristic - Location	Italy

Machine-accessible metadata file describing the reported data: 10.6084/m9.figshare.12515256

## Background & Summary

On January 30, 2020, the COVID-19 outbreak was declared a Public Health Emergency of International Concern by the WHO and since then, it has infected more than 3 million individuals spreading in almost every country in the world, reaching pandemic proportions^1^. As of mid-April, Italy is one of the countries most severely affected by the pandemic, with a death toll that surpassed 20,000.

To contain and mitigate the COVID-19 epidemic, Italy has been the first European country to implement unprecedented measures to restrict individual mobility, and to promote social distancing, with the aim of interrupting transmission of the SARS-CoV-2 virus. Following the detection of the first cluster of COVID-19 cases in Lombardy, on 21 February 2020, the government adopted an increasing number of orders, ranging from school and university closures, limits placed on large social gatherings, closure of bar and restaurants, and a national stay-at-home order. On March 12, 2020, all non-essential business and services have been closed, effectively putting the country under lockdown. Similar Non-Pharmaceutical Interventions (NPIs) have been adopted in several other countries, as they represent the only effective strategy for slowing the spread of the COVID-19 epidemic^2^.

The availability of de-identified and aggregated mobility data has been recognized as a relevant opportunity to quantify the effectiveness of NPIs in promoting social distancing^3,4^. Digital traces collected from smartphone users have been used to quantify the changes in mobility during the lockdown in Wuhan, China^5,6^. Similar studies have been conducted in the United States and other European countries. Moreover, large tech companies such as Google and Apple have published periodic mobility reports based on the analysis of anonymised location data collected through their services^7,8^.

To assess the impact of the NPIs imposed by Italian authorities in response to the COVID-19 epidemic on mobility, we analyzed a de-identified, large-scale dataset from a location intelligence and measurement platform, Cuebiq Inc.

From the original location data, we derive three epidemiologically relevant metrics of mobility and proximity which are reported as 3 different data records: (i) the daily origin-destination matrices measuring users’ movements between Italian provinces; (ii) the weekly users’ average radius of gyration by province, capturing the extent of individual movements; (iii) the daily average degree of users’ proximity network, capturing the level of social distancing by province. All these metrics are computed by aggregating the original data sources in space and time in order to comply with privacy principles that ensure users cannot be re-identified, even indirectly, from the data.

## Methods

### Pre-processing of the original data sources

Location data is provided by Cuebiq Inc., a location intelligence, and measurement platform. Through its Data for Good program^9^, Cuebiq provides access to aggregated and privacy-safe mobility data for academic research and humanitarian initiatives. This first-party data is collected from anonymized users who have opted-in to provide access to their location data anonymously, through a GDPR-compliant framework.

Location is collected anonymously from opted-in users through a Software Development Kit (SDK) included in partner smartphone applications. At the device level, iOS and Android operating systems combine various location data sources (e.g. GPS, wifi, beacons, network) and provide geographical coordinates with a given level of accuracy. Location accuracy is determined by the device and is variable, but can be as accurate as 10 meters. Temporal sampling of de-identified users’ location is also variable and dependent on app/OS characteristics and on user behavioral patterns, but it has a high-frequency overall. The basic unit of information we process is an event of the form (anonymous hashed user id, time, latitude, longitude, accuracy, device) which we call a *stop* in the remainder. The *duration*, Δ*t*_*i*_, of a stop *i* is defined as the time elapsed between the stop *i* and the following stop *i* + 1.

**Fig. 1:**
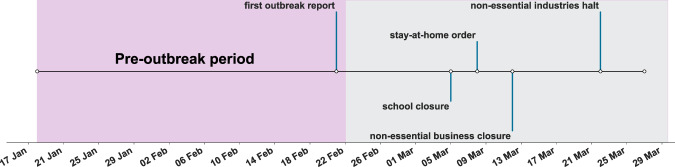
Timeline of data collection and major events in the early phase of the COVID-19 outbreak in Italy.

We curated data that were collected every day for 13 consecutive weeks, from 18 January 2020 to 17 April 2020, included. The timeline displayed in Fig. 1 describes some relevant events that took place in Italy in the early phase of the outbreak. The official report of the first COVID-19 outbreak in the town of Codogno, in the province of Lodi, was announced on February 21, 2020. We define the time window before this date as the *pre-outbreak period*. During the outbreak, after February 21, a number of orders were issued to limit individual mobility and increase social-distancing, as indicated by the labels on the chart.

**Fig. 2:**
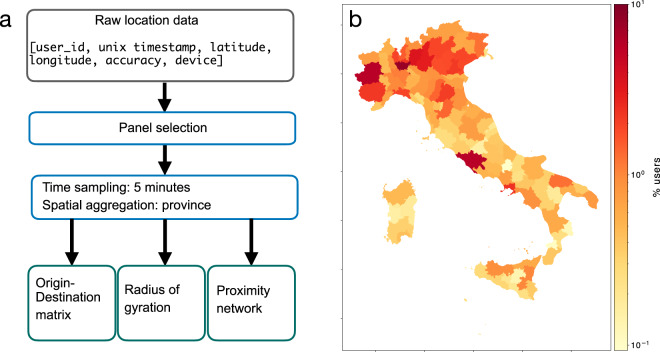
Workflow of the data processing pipeline (**a**). Spatial distribution of the user panel by province (**b**).

The workflow of Fig. 2a outlines the processing pipeline used to generate the data records. We first select a panel of users, based on the condition of being active during the pre-outbreak period and during the week between 22 and 28 February 2020, i.e. for whom at least one data record has been collected both before and after the outbreak. We then select all data records from such panel of users, from 18 January to April 17, for further analysis. Such selection leads to a panel of 167,286 users and a total of about 200 million data points.

We process the basic unit of information in two ways:

- **Time aggregation**. We remove short-time dynamics by aggregating the data over 5-minute windows, dividing the UNIX Epoch time of each record, time, by 300 seconds plus rounding. The location of a user in a 5 minutes interval (i.e., once the aggregation is done) is taken as the geometric center of all recorded user’s (lat, lon) pairs during that interval. After the temporal sampling, the minimum duration of a user’s stop *i* is Δ*t*_*min*_ = 5 minutes.
- **Spatial aggregation**. We assign each user to a province of residence (“home location”) defined as the most visited province in the pre-outbreak period. We assume that home location is the most frequently visited night time location, as it is usually done in the literature^10^. Thus we define the home province to be the province where a user has spent most of the time within the time interval 00:00–6:00, between 18 January and 21 February 2020. We consider all the stops whose duration has an intersection with the interval 00:00–06:00 (e.g., stops starting at 9 pm and ending at 00:30, or the next day after 6 am).

**Fig. 3:**
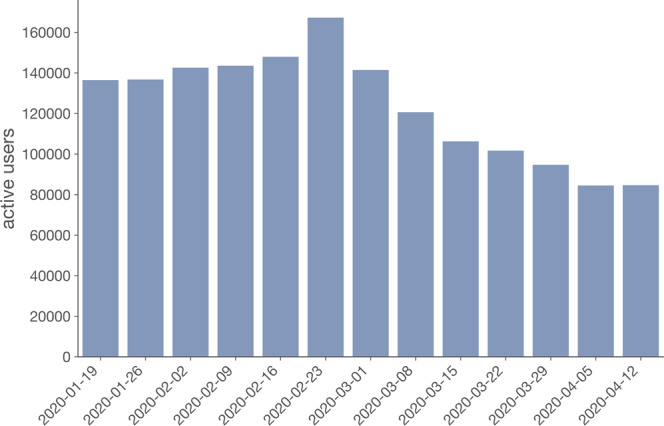
Weekly number of active users in the panel under study. Date on the x-axis refers to the first Sunday of a week.

The users sample size varies in space and time. The spatial distribution of users’ by home province is shown in Fig. 2b A higher proportion of users is present in the provinces of Northern Italy. Also, the number of active users is not constant over time, as shown in Fig. 3. As the outbreak progresses, the weekly number of active users in our sample decreases to a minimum of 84,699 active users in the week of 11–17 April.

### Mobility networks

We generate weighted daily origin-destination (OD) matrices capturing movements between Italian provinces, by considering the users’ individual trajectories and by assigning each time-ordered stop to a province. In the trajectory of a user we consider as stops only the provinces where a user remained for more than 1 hour. This is done to remove transit visits (if a user is for instance travelling by train, he might cross several provinces without stopping).

Every time a user moves from province *i* to province *j* we add +1 to the corresponding connection (*i*, *j*). If a user never moves outside province *i* in a day, we add +1 to the entry (*i*, *i*) of the matrix.

We further normalize the OD matrix by rows, so that each entry of the matrix (*i*, *j*) represents the fraction of movements made by users traveling from province *i* to province *j*, each day.

### Radius of gyration

The radius of gyration of a user, *r*_*g*_, provides a measure of the spatial range of a users’ mobility patterns^11^.

It is defined as:

1 $${r}_{g}=\frac{1}{L}\sqrt{\mathop{\sum }\limits_{i=1}^{L}{({{\bf{r}}}_{{\bf{i}}}-{{\bf{r}}}_{{\bf{cm}}})}^{2}}$$

where *L* is the full set of stops made by a user over a given time frame, **r**_**i**_ is the vector of coordinates of stop *i* and **r**_**cm**_ is the vector of coordinates of the center of mass, weighted by the duration of each stop Δ*t*_*i*_.

We compute the radius of gyration for each user on a weekly basis, so that *L* in Eq. 1 represents the set of stops made by a user during a week. We then compute descriptive statistics of the distribution of the radius of gyration by users’ home province.

### Proximity network

The average contact rate, or the number of unique contacts made by a person on a typical day is a fundamental quantity to model and understand infectious disease dynamics^12^. We evaluated the effect of NPIs on the proximity of our users’ sample, by defining a proxy of the potential encounters each anonymous user could have in one hour. To this aim, we built a proximity network among users based on the locations they visited and the hour of the day when these visits occurred.

**Fig. 4:**
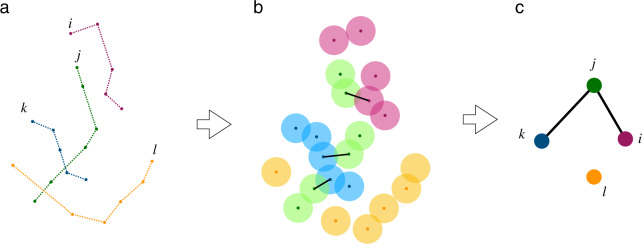
Workflow to build the users’ proximity network. Users’ trajectories are generated (**a**). A circle of fixed radius is drawn around each user’s stop (**b**). The proximity network is defined by the intersecting circles of different users (**c**).

 Fig. 4 describes the workflow used to build the proximity network. We first collect all the positions of all users in a given province within time windows Δ*t* = 1 hour (from 00:00 to 23:59), as shown in Fig. 4a. We then create a disk of radius *R* = 50 m around each stop of the users (Fig. 4b). Finally, if two disks of a pair of different users intersect during the same time window, we place a link between the two users in the resulting network. Multiple links are counted only once every hour.

**Fig. 5:**
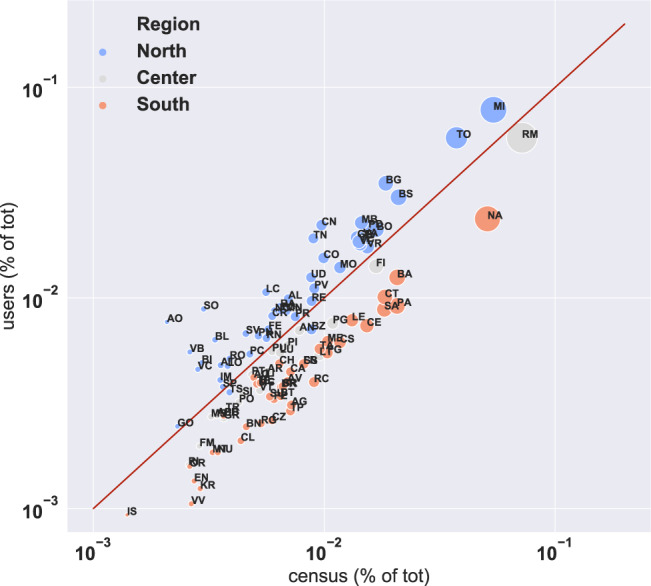
Scatterplot of the number of users assigned to each Italian province against the resident population reported by the Italian census in each province, as a fraction of the totals. Color code correspond to the three main geographic areas of Italy: North, Center, South.

We measure the mean hourly network degree as 〈*k*〉 = 2*E*/*N*, where *E* is the number of edges and *N* is the total number of nodes in the network, including those with *k* = 0. The mean daily degree is obtained by averaging all the 24 values of 〈*k*〉 measured in a day, in a given province.

It is important to remark that this is not a close-range contact network. Rather, it captures a looser notion of social mixing at the chosen spatial and temporal scales. A link between two nodes indicates the possibility that the corresponding individuals have had a close-range encounter during a given day. Because of the non-uniform spatial sampling, it is also important to notice that raw values of 〈*k*〉 are not comparable across provinces. Provinces with a smaller users sample are characterized by lower values of 〈*k*〉. Instead, relative variations of 〈*k*〉 between the pre-outbreak and the outbreak period are informative of the effects social distancing on our users’ sample.

## Data Records

A static copy of the dataset has been uploaded on Figshare^13^. A live version of the data record, which will be kept up-to-date with new estimates, can be downloaded from the Humanitarian Data Exchange: https://data.humdata.org/dataset/covid-19-mobility-italy.

The data record is structured into 4 comma-separated value (CSV) files, as follows:

- id_provinces_IT.csv. Table of the administrative codes of the 107 Italian provinces. The fields of the table are:
  - COD_PROV is an integer field that is used to identify a province in all other data records;
  - SIGLA is a two-letters code that identifies the province according to the ISO_3166-2 standard (https://en.wikipedia.org/wiki/ISO_3166-2:IT);
  - DEN_PCM is the full name of the province.
- OD_Matrix_daily_flows_norm_full_2020_01_18_2020_04_17.csv. The file contains the daily fraction of users’ moving between Italian provinces. Each line corresponds to an entry of matrix (*i*, *j*). The fields of the table are:
  - p1: COD_PROV of origin,
  - p2: COD_PROV of destination,
  - day: in the format yyyy-mm-dd.
- median_q1_q3_rog_2020_01_18_2020_04_17.csv. The file contains median and interquartile range (IQR) of users’ radius of gyration in a province by week. Each entry of the table fields of the table are:
  - COD_PROV of the province;
  - SIGLA of the province;
  - DEN_PCM of the province;
  - week: median value of the radius of gyration on week week, with week in the format dd/mm-DD/MM where dd/mm and DD/MM are the first and the last day of the week, respectively.
  - week Q1 first quartile (Q1) of the distribution of the radius of gyration on week week,
  - week Q3 third quartile (Q3) of the distribution of the radius of gyration on week week,
- average_network_degree_2020_01_18_2020_04_17.csv. The file contains daily time-series of the average degree 〈*k*〉 of the proximity network. Each entry of the table is a value of 〈*k*〉 on a given day. The fields of the table are:
  - COD_PROV of the province;
  - SIGLA of the province;
  - DEN_PCM of the province;
  - day in the format yyyy-mm-dd.

ESRI shapefiles of the Italian provinces updated to the most recent definition are available from the website of the Italian National Office of Statistics (ISTAT): https://www.istat.it/it/archivio/222527.

## Technical Validation

### Geographic representativeness

We tested the geographic representativeness of our sample by comparing the size of our users’ sample against the population reported by the 2011 census in each province.  Fig. 5 shows a scatterplot of the two populations as percentage of the total, by province. The size of the dots is proportional to the total census population size. Overall, our user base over-represents the population in the North of Italy, while it under-represents the Center and the South. The Pearson population-weighted correlation coefficient between the two datasets is *r* = 0.85, *p* < 10^−5^.

### Sensitivity analysis on the proximity network

We performed a sensitivity analysis on the parameters used to generate the proximity network, to test the robustness of our results. The sensitivity analysis on the results obtained by different values of *R* and the chosen temporal bin, Δ*t*, is reported in Table 1. We compute the relative reduction of the average degree during the first 4 weeks of outbreak, 〈*k*〉_*outbreak*_, with respect to the average degree over the 5 weeks preceding the outbreak (〈*k*〉_*pre*–*outbreak*_) and we check how the reduction changes with respect to Δ*t* and *R*.

**Table 1:** Sensitivity analysis on *R* and Δ*t* to generate the proximity networks.

*R* (m)	Δ*t* (min.)	〈*k*〉_*pre*–*outbreak*_	〈*k*〉_*outbreak*_	〈*k*〉 relative reduction
50	60	0.123	0.080	35%
	30	0.061	0.041	33%
	15	0.031	0.021	32%
25	60	0.042	0.027	36%
	30	0.02	0.014	30%
	15	0.01	0.007	30%

**Table 2:** Pearson’s correlation coefficient between time-series of mobility reductions reported by Google^7^ and daily time-series of the average degree 〈*k*〉 of the proximity network.

Google mobility metric	Pearson *r*	p-value
retail and recreation	0.98	p < 10^−6^
grocery and pharmacy	0.88	p < 10^−6^
parks	0.97	p < 10^−6^
transit stations	0.97	p < 10^−6^
workplaces	0.95	p < 10^−6^

As expected, smaller values of *R* and Δ*t* lead to sparser networks, but the overall reduction observed from the pre-outbreak period is stable across different spatio-temporal scales.

### Comparison with alternative data sources

As a quality control of our measures, we compared the proximity metric derived from our sample with the mobility data reported by Google^7^ at the national level.

Google Community Mobility Reports provide daily time-series of mobility changes across different categories of places such as retail and recreation, groceries and pharmacies, parks, transit stations, workplaces, and residential.

We used the period between January 18 and February 15 as our baseline and compared the daily time-series of the reduction average degree of the proximity network, 〈*k*〉, at the national level with the mobility reduction time-series provided by Google between February 16 and March 27, 2020.

As shown in Table 2, the temporal variations of 〈*k*〉 are highly correlated with the mobility reductions reported by Google in Italy across all sectors, indicating the same temporal trend is captured by both data sources.

## Usage Notes

These data are useful for investigating the effects of different types of social distancing interventions on population mobility, and as inputs for mechanistic models of disease spread^4^. On the one hand, these data can be used to fine-tune epidemic computational models, by integrating the observed behavioural changes, allowing for *a-posteriori* estimates of the impact of interventions on the spatial spread of the COVID-19 epidemic. On the other hand, these data can represent a benchmark to evaluate the effects of relaxation plans as mobility restrictions will be gradually lifted in Italy and elsewhere.

The origin-destination matrix are necessary both to epidemic metapopulation models that simulate the disease spread among structured sub-populations where the pathogen invasion is driven by individual mobility^14,15^, and to econometric models that measure the economic impact of mobility restrictions across different business sectors. Similarly, temporal variations of the radius of gyration and the average degree of the proximity network can be mapped onto an effective force of infection in epidemic compartmental models.

Such studies are of crucial importance to test in-silico scenarios that can inform decision makers about the economic costs and the public health impact of mitigation policies. Finally, these data represent a benchmark to evaluate the impact of policies in a highly developed economy, and thus can be used in the future to compare the effects of different policies or measuring the compliance of the population to similar ones.

When using the data for epidemic or economic modeling, a few limitations must be acknowledged. The first is that the data is limited to users who have opted-in for anonymously sharing their location with Cuebiq. Geographically, provinces in the North of Italy are over-represented with respect to the Center and the South. Also, the users’ sample can not be considered to be demographically representative of the Italian population by age or gender, as such information is not available. Finally, the user base changes over time and some demographic groups could become more or less represented at different points in time. Temporal variations of the user base have several causes. First, users can opt-out for anonymously sharing their location at any time. Second, recording of a user’s location is triggered by using specific apps, or through geofencing, and these mechanisms will depend on the phone operative system (Android or iOS). Therefore, we might lose visibility of users who are stationary for a long time, or who don’t use a given app for an extended period of time during lockdown.

## Data Availability

All data records were generated using code developed in Python 3^16^. The code is available upon request from the corresponding author.
